# A Participatory Artificial Intelligence Driven Shift‐Scheduling Application for Improving Sleep Among Shift‐Working Caregivers: A 4‐Month Non‐Randomised Controlled Study With Cross‐Over Design

**DOI:** 10.1111/jsr.70144

**Published:** 2025-07-17

**Authors:** Tomohide Kubo, Shun Matsumoto, Yuki Nishimura, Hiroki Ikeda, Shuhei Izawa, Fumihiko Sato

**Affiliations:** ^1^ Research Center for Overwork‐Related Disorders National Institute of Occupational Safety and Health Kawasaki Japan; ^2^ SynCube Co. Ltd. Tokyo Japan

**Keywords:** artificial intelligence, caregiver, occupational‐fatigue counselling approach, shift work, wearable device, worktime control

## Abstract

Here, we examine the effectiveness of a participatory artificial intelligence (AI)‐driven shift‐scheduling mobile application (which reflects the local improvement needs in shift scheduling) in improving the sleep quality of shift‐working geriatric caregivers. Thirty‐five shift‐working geriatric caregivers participated in this 4‐month cross‐over interventional study. Half of the participants in the first 2 months followed the intervention schedule created by the AI‐driven shift‐scheduling mobile application, while the remaining participants followed the manually created control schedule. The improvement needs in shift scheduling, derived from occupational‐fatigue counselling, were as follows: avoiding backward rotating shifts, reducing consecutive shifts, extending shift intervals and ensuring a day‐off after a night shift. Sleep phases were evaluated using a ring‐type sleep tracker. The effectiveness of the intervention was examined using three‐way multilevel analyses (condition × shift × time). Deep sleep (N3) and rapid eye movement sleep were significantly more pronounced in the intervention condition compared with the control condition (*p* = 0.016, *p* = 0.046, respectively). However, no significant differences were detected for other outcomes. Moreover, we examined how shift combinations affected sleep outcomes. As a result, two consecutive late shifts and backward rotating shifts significantly deteriorated sleep quality and length (all *p* < 0.05). Our findings suggest that the shift‐scheduling app reduced the backward shift rotations, resulting in significantly better sleep outcomes than from manual schedule creation. However, the magnitude of reduction in backward rotating shifts was not so remarkable. Therefore, the positive outcomes can also be attributed to enhanced employees' working time control by reflecting the local improvement needs.

**Trial Registration:** UMIN Clinical Trials Registry: UMIN000048495

## 1 Introduction

Shift workers often face greater health and safety risks than those working normal hours (Wong et al. 2019). Empirical data shows that working shifts and health and safety issues (Wong et al. 2019; Härmä et al. 2020; Marquié et al. 2015; Sharma et al. 2017; Stevens et al. 2014) are interlinked. Establishing sustainable working hours is critical in ensuring a healthy working environment (Härmä et al. 2024). However, developed countries with a large ageing population (Gerland et al. 2022) require a strong workforce engaged in geriatric care. Geriatric caregivers cannot avoid shift work, as they must care for older adults living in nursing homes. Moreover, in future, the need for geriatric caregivers is expected to increase in response to an increased ageing rate. Meanwhile, literature on healthy and safe shift‐work scheduling for geriatric caregivers is lacking compared with that on shift scheduling for nurses, though some studies focused on geriatric caregivers (Nabe‐Nielsen et al. 2010, 2009). This dearth in studies may have been caused by the assumption that observations from studies conducted on the requirements of nurses can be extrapolated to those of other caregivers.

However, the working conditions of nurses and other shift‐working geriatric caregivers are quite distinct, despite sharing some similarities. For example, approximately 90% of the workforce in Japan adopted a two‐shift system with a 16‐h night shift (Japan Federation of Medical Worker's Unions 2024). A previous study suggested an association between long work shifts (over 12‐h) and adverse outcomes (Sallinen and Kecklund 2010). Moreover, it is a common practice in Japan to randomly assign workers to different shift schedules and lengths each week. Besides, the salary level of geriatric caregivers can be much lower than that of the nurses (Japan Ministry of Health Labour and Welfare 2022). Such differences can be linked to fatigue and stress levels in nurses and other shift‐working geriatric caregivers. Therefore, empirical data specific to shift‐working geriatric caregivers are required to optimise their working conditions.

Work schedule is a critical factor contributing to the development of occupational‐fatigue in workers (Techera et al. 2016). Thus, it is expected that interventions affecting work schedule will help manage occupational‐fatigue. Therefore, here we focus on examining the effectiveness of modifying shift schedules of shift‐working geriatric caregivers on sleep and fatigue in geriatric caregivers. However, modifying shift schedules without taking individual work routines into consideration will limit their success in reducing fatigue and improving sleep. Increasing the employees' influence over working hours (i.e., worktime control) is reported to improve health outcomes (Ala‐Mursula et al. 2004). Conversely, the potential risk of sleep disturbance was higher when the employees had a low worktime control level in combination with shift work or informal caregiving (i.e., caring for an ill or disabled child or other members of a person's social network) (Virtanen et al. 2021). Therefore, to enhance the employees' worktime control, we adopted an occupational‐fatigue counselling approach in which inputs from interactions between researchers and local employees were used. The inputs from this interactive participatory process reflected the working realities and accurately pinpointed the improvements needed in shift scheduling, which could counter the occupational‐fatigue in the employees (Kubo et al. 2022). Conversely, it would be challenging for a shift planner to manually make a shift schedule that matches all individual needs. Therefore, it is reported that using artificial intelligence (AI) would be beneficial in making a fair and satisfying shift schedule (O'Callahan et al. 2024). Based on these backgrounds, we developed an original AI‐driven shift‐scheduler mobile application (app) that could automatically create an individualised workplace‐customised shift schedule using inputs from occupational‐fatigue counselling. This 4‐month non‐randomised interventional cross‐over study aimed to examine the effect of our AI‐driven shift‐scheduling mobile app on sleep quality and fatigue rates among the participating geriatric caregivers. Since sleep plays an essential role in recovery from work (Hetland et al. 2022), we objectively measured sleep quality and quantity using a ring‐type sleep tracker to examine the interventional effect. In addition, we aimed to investigate how shift patterns, such as backward rotation or consecutive shifts, affect sleep outcomes.

## 2 Methods

### 2.1 Participants

The inclusion criteria for participants in this study were (1) age: 20–50 years old, (2) no previous complaints of sleep disorders and (3) currently working on a shift schedule. Finally, 35 shift‐working geriatric caregivers from two local areas in Hokkaido, Japan, with similar characteristics concerning working conditions, were enrolled. The descriptive statistics for their characteristics (mean and standard deviation) are as follows: age: 41.1 ± 12.9 years old, work experience: 10.2 ± 5.9 years, body mass index (BMI): 23.6 ± 4.4 and one‐way commute time: 16.5 ± 10.6 min. Twenty‐five of the 35 participants were female, of whom 24 were single, and 3 had a child younger than 12 years old. SynCube Co. Ltd., Japan (the author F.S. is the president of this company), selected the workplace and recruited the participants. The local review board reviewed and approved the study protocol (2022N‐1‐6). We registered this study in the UMIN Clinical Trials Registry (UMIN000048495). We obtained written informed consent from all participants, and all participants were paid to participate.

### 2.2 Study Design

**FIGURE 1:**
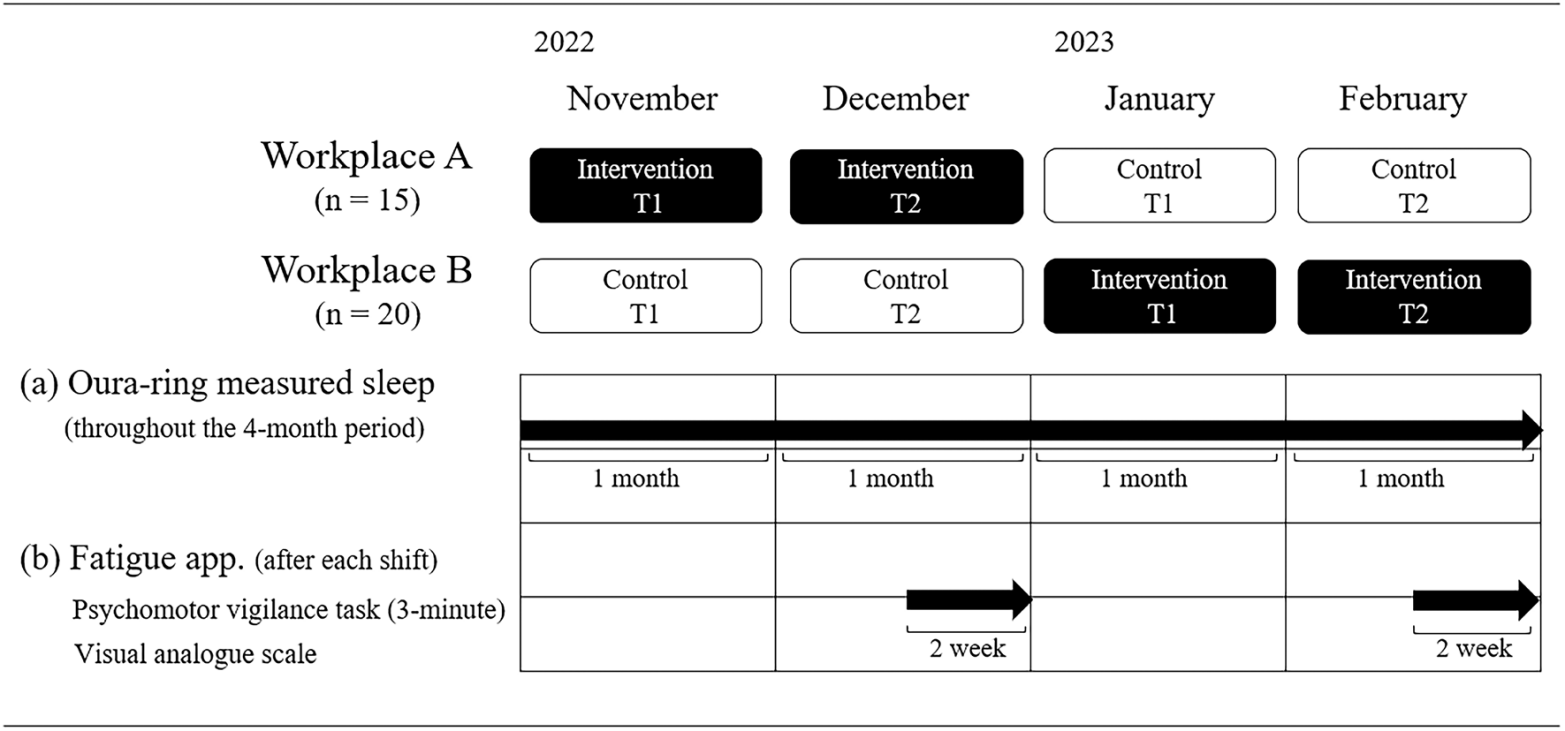
Scheme of study design; the first (T1) and the second halves (T2) of the intervention or control period.

We used a 4‐month‐long non‐randomised cross‐over intervention study design (Figure 1). In the first 2 months of the study, half of the participants were asked to follow the ‘intervention’ shift schedule created using the AI‐driven shift‐scheduling app (Synchroshift, SynCube Co. Ltd., https://www.youtube.com/watch?v=FDRGtUUfn‐0), while the remaining half of the participants followed the manually created ‘control’ shift schedule. In the intervention condition, all the employees, including the participants, worked under the shift schedule created by the app. Regarding the control of shift schedules, the managers in the workplace created a shift schedule based on their knowledge and experience. After 2 months, the conditions were switched. Also, the first month during control or intervention was designated as T1, while the second month was denoted T2. The rationale for splitting each condition into 2 months was the hypothesis that it would be a few months before the effectiveness of this intervention would be observed. Basically, the shift schedules were as follows: early (6:00 AM–3:00 PM), day (8:00 AM–5:00 PM), late (11:00 AM–8:00 PM) and night shifts (4:00 PM–10:00 AM).

### 2.3 Intervention Shift Schedules

During the intervention period, the participants were assigned to work under the shift schedule created by the AI‐driven shift‐scheduling mobile app. Approximately 1 year before this study commenced, we carried out occupational‐fatigue counselling (Kubo et al. 2022) for 25 geriatric caregivers who were randomly selected volunteers from the target population prior to the study design elaboration to collect the information necessary for desirable shift scheduling geared towards fatigue recovery. Specifically, we interviewed each geriatric caregiver with at least three questions during an almost 1‐h session: (1) What shift combination could make you tired? (2) When do you feel tired on duty? (3) What improvements could be effective in recovering from fatigue in your workplace? Based on the information, we discussed the contents of improvement with the managers' side in terms of effectiveness and feasibility (the improvement needs were shown in Section 3). At the time, we provided some knowledge and evidence regarding shift work for the managers (not including the shift planners). The AI‐driven shift‐scheduling app created a shift schedule after incorporating these requirements using meta‐heuristic algorithms based on integer programming and a tab search method.

### 2.4 Measurement

#### 2.4.1 Working Hours and Days‐Off

During the period of the study, the data on working hours were objectively calculated by the pay‐roll records in the involved workplace. The monthly number of days‐off was calculated using these data. We measured those data to determine whether the effectiveness of the intervention on the shift schedule could be derived from their reduced working hours and increased number of off days, in addition to changing the shift schedule.

#### 2.4.2 Sleep

We measured a day‐to‐day variation in six sleep outcomes (i.e., total sleep time [TST], sleep efficiency, deep sleep time [i.e., N3], rapid eye movement [REM] sleep time and sleep latency) by using a ring‐type sleep tracker device (i.e., Oura ring Gen3, Ōura Health Oy, Finland) for 4 months. The participants were instructed to charge their ring‐type sleep tracker during their daily bath, over the whole study period. The accuracy of this device in qualitatively and quantitatively evaluating sleep has been validated in earlier polysomnography (PSG) studies (Willoughby et al. 2024; Svensson et al. 2024).

#### 2.4.3 Fatigue

We used the smartphone‐based fatigue app (named Fatigue Checker) developed by our research team (Kubo et al. 2018; Ikeda et al. 2023). The participants read the quick‐response code printed in their instructions with their smartphone to perform the measurements. The fatigue checker could easily and effectively measure fatigue‐related psychological (Visual Analogue Scale [VAS]) and behavioural (psychomotor vigilance task [PVT]) outcomes. The participants were instructed to evaluate their current sleepiness and fatigue on a 100‐point scale, respectively. The value ranged from 0 points (e.g., not at all sleepy) to 100 points (e.g., extremely sleepy). Meanwhile, the outcomes of a 3‐min PVT (PVT‐B; Basner et al. 2011) were defined as behavioural outcomes. PVT‐B presented a digital counter on the screen at irregular interstimulus intervals, ranging from 1 to 4 s. The participants were asked to respond to a digital counter as quickly as possible. In addition, the first page of the guidelines visualised on their smartphone's screen enabled explaining how to ban looking away from a screen during the test period. Mean reaction times and lapses (defined as lasting longer than 355 ms) were calculated as the outcomes to evaluate their objective performance. In the intervention and control conditions, the fatigue checker was activated in the break room after each shift for a 2‐week period.

### 2.5 Data Analysis

Data regarding shift scheduling improvement needs were recorded using the AI‐driven shift‐scheduling app. Moreover, the data were compared between the intervention and control conditions using the Wilcoxon signed‐rank test. Data on the length of working hours and the number of days‐off were evaluated by the two‐way (condition [intervention, control] × time [T1, T2]) multilevel analysis. Sleep data were analysed by using a three‐way (condition [intervention, control] × shift [day, early, late, night and day‐off] × time [T1, T2]) multilevel method to evaluate the effect of the intervention on these outcomes. In contrast, fatigue‐related outcomes were assessed by implementing a two‐way multilevel analysis (condition [intervention, control] × time [T1, T2]), because it was difficult to gather the data, including all types of shifts within a 2‐week period. These analyses covered conditions, shifts and periods of time as fixed factors and entered each participant as a random factor in the linear mixed‐effect model. The significant main effect of the shift was evaluated using the post hoc Bonferroni test. Moreover, our purpose was to examine how shift combination affected sleep quality and quantity. Furthermore, we focused on six representative shift combinations, as follows: (1) early‐to‐night shift (EN), (2) late‐to‐night shift (LN), (3) early‐to‐early shift (EE), (4) late‐to‐late shift (LL), (5) night‐to‐day‐off to day‐off (NDD) and (6) backward rotation (i.e., day‐to‐early, late‐to‐early, late‐to‐day, night‐to‐early, night‐to‐day and night‐to‐late shifts). Monthly mean values regarding sleep outcomes were calculated to examine their association with the monthly number of shift combinations. Then, multivariate mixed‐model regression analysis was conducted to reveal which shift combinations could have stronger effects on sleep outcomes. Shift combinations, conditions and time were considered fixed factors, while the participants were outlined as a random. Since our main attention was on the association between shift combinations and sleep outcomes, Table 1 showed only the statistical results of the comparative analysis of shift combinations. In all the analyses except for those on the data on working hours and days‐off, age, sex, marital status (married/single), workplace (A/B), career, BMI, alcohol intake (yes/no), exercise (yes/no) and infection of COVID‐19 (yes/no) were used as covariates. Since we considered that the participants who were infected with COVID‐19 would have more stress than those who were not, we obtained the data from the manager and used these as covariates. The analysis was carried out by applying IBM SPSS Statistics version 26 (IBM Corp., Armonk, NY, USA), where *p* < 0.05 was regarded as statistically significant.

**TABLE 1:** Association between the monthly number of six shift combinations and the monthly mean value of sleep outcomes.

	*β*	SE	*p*	95% CI
Total sleep time (h)				
Intercept	4.514	1.335	0.002	1.767/7.262
EN	−0.033	0.076	0.660	−0.184/0.117
LN	0.090	0.059	0.127	−0.026/0.206
EE	−0.111	0.127	0.384	−0.364/0.141
LL	**−0.281**	**0.135**	**0.040** *	**−0.55/−0.013**
NDD	−0.036	0.078	0.643	−0.191/0.118
Backward rotation	−0.049	0.080	0.542	−0.207/0.109
Sleep efficiency (%)				
Intercept	71.441	11.539	0.000	47.674/95.208
EN	−0.240	0.720	0.739	−1.669/1.188
LN	0.331	0.556	0.553	−0.772/1.434
EE	−1.384	1.208	0.255	−3.782/1.013
LL	**−2.678**	**1.285**	**0.040** *	**−5.229/−0.128**
NDD	−0.235	0.740	0.752	−1.703/1.234
Backward rotation	−0.752	0.755	0.322	−2.25/0.746
Deep sleep [N3] (min)				
Intercept	99.095	21.755	0.000	54.435/143.755
EN	−0.878	1.590	0.582	−4.033/2.277
LN	1.204	1.225	0.328	−1.227/3.635
EE	−1.616	2.661	0.545	−6.895/3.663
LL	**−5.804**	**2.835**	**0.043** *	**−11.43/−0.178**
NDD	−1.350	1.632	0.410	−4.589/1.889
Backward rotation	−2.320	1.663	0.166	−5.618/0.979
REM sleep (min)				
Intercept	34.921	33.791	0.311	−34.563/104.405
EN	−0.928	1.475	0.530	−3.856/2
LN	1.852	1.141	0.108	−0.413/4.118
EE	−1.162	2.484	0.641	−6.093/3.769
LL	−4.103	2.635	0.123	−9.336/1.131
NDD	−0.701	1.517	0.645	−3.713/2.31
Backward rotation	−0.643	1.554	0.680	−3.728/2.441
Sleep latency (min)				
Intercept	10.581	3.471	0.005	3.461/17.7
EN	0.233	0.254	0.360	−0.27/0.736
LN	0.353	0.196	0.075	−0.036/0.741
EE	0.155	0.426	0.717	−0.69/0.999
LL	−0.151	0.453	0.741	−1.05/0.749
NDD	0.093	0.261	0.722	−0.425/0.611
Backward rotation	**0.584**	**0.266**	**0.030** *	**0.057/1.111**

*Note*: Age, sex, marital status, workplace, career, BMI, alcohol intake, exercise and COVID‐19 infection were introduced as covariates. Basically, their shift schedules were as follows: day shift (8:00 AM–5:00 PM), early shift (6:00 AM–3:00 PM), late shift (11:00 AM–8:00 PM) and night shift (4:00 PM–10:00 AM). Values in bold indicate signifcant diferences.

Abbreviations: CI, confidence interval; EE, early‐to‐early shift combination; EN, early‐to‐night shift combination; LL, late‐to‐late shift combination; LN, late‐to‐night shift combination; NDD, night‐to‐day‐off to day‐off combination.

* *p* < 0.05.

## 3 Results

### 3.1 Shift Changes Outlined by AI‐Driven Shift‐Scheduling App

The occupational‐fatigue counselling revealed four shift scheduling improvement needs, namely, (1) avoiding backward rotating shift, (2) reducing consecutive shifts, (3) extending shift intervals and (4) ensuring days‐off after a night shift. We observed that the AI‐driven shift‐scheduling app modified the shift schedules for all participants on all these four counts as reflected by the differences in their number of occurrences in intervention and control conditions (intervention vs. control, median and interquartile range): (a) backward rotating shift (0.0 [0.0–1.0] vs. 1.0 [1.0–2.0]), (b) consecutive shifts (0.0 [0.0–0.0] vs. 0.0 [0.0–0.0]), (c) the extended shift intervals (1.0 [0.0–2.0] vs. 1.0 [0.0–2.0]) and (d) ensured days‐off after a night shift (2.5 [2.0–3.25] vs. 3.0 [2.0–4.0]). However, only the backward rotating shift (*Z* = −2.841, *p* = 0.005) was significantly different between the intervention and control schedules. Thus, overall, our AI‐driven shift‐scheduling app mainly reduced the number of backward rotating shifts.

### 3.2 Objective‐Recorded Working Hours

Statistical analysis showed that the length of working hours and the number of day‐offs were not significantly different in terms of condition, time and interaction. The average and standard error of the mean of working hours were as follows: intervention: T1; 10.9 ± 0.2 h/T2; 11.1 ± 0.2 h, control: T1; 11.1 ± 0.2 h/T2; 11.2 ± 0.2 h, while those of day‐offs were as follows: intervention: T1; 9.6 ± 0.4 h/T2; 10.1 ± 0.4 h, control: T1; 9.9 ± 0.4 h/T2; 9.3 ± 0.4 h.

### 3.3 Sleep

**FIGURE 2:**
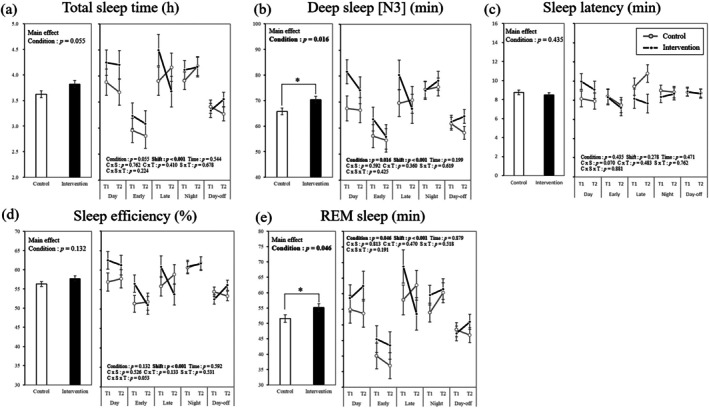
Intervention effect of AI‐driven shift‐scheduling app on sleep outcomes measured by ring‐type sleep tracker. Data represent the estimated marginal mean and standard error of mean. Age, sex, marital status, workplace, career, BMI, alcohol intake, exercise and COVID‐19 infection are shown as covariates.

Figure 2 shows the interventional effects of using an AI‐driven shift‐scheduling app on sleep outcomes. Of the five sleep outcomes studied, significant differences between intervention and control conditions were noticed for N3 and REM sleep (*p* = 0.016 and *p* = 0.046, respectively). Namely, the length of N3 and REM sleep was longer in the intervention condition than in the control. TST showed a similar tendency, though no significant differences were observed (*p* = 0.055). The type of shifts significantly affected four outcomes (TST, N3, sleep efficiency and REM sleep) (Figure S1). Regarding the interaction for each factor, no significant findings were observed (i.e., condition × shift, condition × time and condition × shift × time).

### 3.4 Fatigue

**FIGURE 3:**
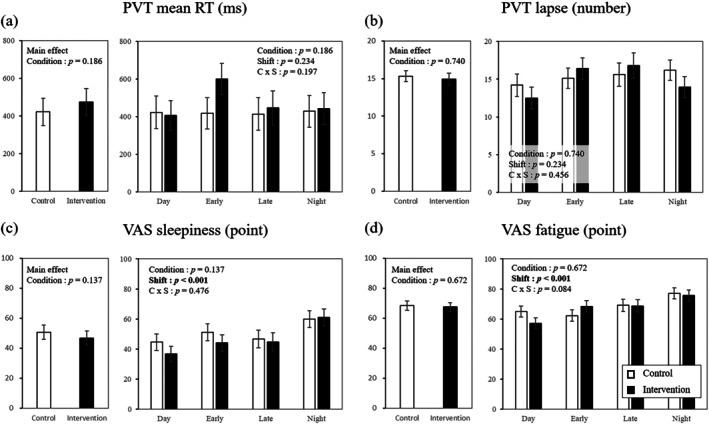
Intervention effect of AI‐driven shift‐scheduling app on behavioural and psychological outcomes measured by smartphone‐based fatigue app. Data display the estimated marginal mean and standard error of mean, and age, sex, marital status, workplace, career, BMI, alcohol intake, exercise and COVID‐19 infection were introduced as covariates.

Figure 3 shows the interventional impact of using an AI‐driven shift‐scheduling app on behavioural and psychological outcomes; the intervention using the AI‐driven shift‐scheduling app did not significantly affect the behaviour and psychological outcomes. Conversely, shift type significantly affected the VAS‐measured sleepiness and fatigue (Figure S2). There were no significant differences regarding interaction in PVT and VAS outcomes.

### 3.5 Shift Combinations and Sleep Outcomes

Table 1 reflects the association between the monthly number of six representative shift combinations and the mean values regarding sleep outcomes. Of the six shift combinations, the significant difference was observed for LL shift combinations in TST, sleep efficiency, and N3 (*p* = 0.040, *p* = 0.040 and *p* = 0.043, respectively). These data suggest that sleep outcomes significantly deteriorated (TST (h); *β* = −0.281, sleep efficiency (%); *β* = −2.678 and N3 (min); *β* = −5.804), as the LL shift's length increased in one occurrence. Also, regarding backward rotation, the significant difference was detected for sleep latency (*p* = 0.030), suggesting that it significantly extended (*β* = 0.584 [min]) with the rise of backward rotation by 1 unit. No significant associations were observed in other outcomes.

## 4 Discussion

The main effect of the AI‐driven shift‐scheduling app in the intervention condition was the reduction of the number of backward rotating shifts compared with those of the control condition. However, no changes were found in the number of working hours and days‐off during the study period. Consequently, significantly better outcomes were seen for N3 and REM sleep in the intervention condition than in the control condition. Conversely, no significant findings regarding the interventional effectiveness were detected for PVT and subjective outcomes. Moreover, this study revealed that specific shift patterns, such as LL shift combination and backward rotating shifts, caused a significant decline in sleep quality and quantity, as objectively measured by a ring‐type sleep tracker.

Here, reduction in backward rotating shift had a positive influence on sleep outcomes. Our findings are consistent with previous reports, which suggest that backward rotating shift could be detrimental (Sallinen and Kecklund 2010; Garde et al. 2020). For instance, an interventional study suggested that changing from backward to forward rotating shift has a positive influence on increasing employee's perceived alertness (Viitasalo et al. 2008). Furthermore, a previous interventional study reported the beneficial effects of a 1‐h delay in shift start times on sleep and performance before the morning shift (Sallinen and Kecklund 2010; Garde et al. 2020). A better sleep outcome was observed in older workers compared to younger workers. Conversely, the magnitude of reduction in backward rotating shifts was not so remarkable in this study. Nonetheless, the positive influence observed in this study could be related to the participants' age (i.e., the average age was in the 40s). In other words, the positive influence may have been greater in older workers compared to younger workers, even though the actual reduction in backward rotation was not large. Moreover, the modified shift schedule was created based on the improvement needs derived from the occupational‐fatigue counselling approach targeted for local employees. Therefore, this intervention could contribute to enhancing the broadly defined ‘employee's worktime control’. In fact, shift workers who had higher worktime control reported fewer complaints of sleep disturbances than those who did not (Sallinen and Kecklund 2010; Garde et al. 2020). Hence, the synergistic favourable impact of avoiding backward rotating shifts and increasing worktime control might be observed for sleep outcomes.

Notably, N3 and REM sleep lengths increased significantly in the intervention condition compared with those in the control condition. Both N3 and REM sleep play an important role in fatigue, sleepiness and stress (McCarter et al. 2022; van der Helm et al. 2011). Recently, a 15‐year‐long cohort study showed that very low REM sleep durations could be associated with a greater risk of several‐cause mortality (including cardiovascular or cancer deaths) (Leary et al. 2020). However, in our study, the N3 and REM sleep lengths were not markedly increased in magnitudes (i.e., N3; about 5 min/REM; about 4 min). Although statistically different, the changes in N3 and REM sleep could be biologically insignificant. Namely, the magnitude of increase in N3 and REM sleep could not enhance PVT performance and VAS score. Contrastingly, intervention comprising repeated exercise—a four‐time repeat of 40‐min aerobic workout at 40% of maximal oxygen intake—increased duration of N3 by approximately 30 min above the baseline (Aritake‐Okada et al. 2019). However, the on‐duty period intervention in our study achieved approximately 5 min increase in N3 and REM, without any off‐duty intervention. Namely, those benefits were afforded by only changing the shift schedule, whereas the participants were not required to exert an extra effort during the off‐duty period. Moreover, as the AI‐driven shift‐scheduling app did not significantly change the number of working hours and days‐off, better sleep outcomes were believed to be achieved by changing the shift schedule alone. Therefore, our intervention strategy is more feasible than the exercise intervention. Furthermore, the effect of the intervention will emerge as significant if the positive effects in our study are long‐lasting. In the next step, examining whether this intervention could help reduce the number of sick calls associated with intervened schedules is important.

Our next objective was to determine the detrimental shift patterns, which negatively affect sleep outcomes. As shown in Table 1, LL shift combination and backward rotation significantly correlated with shorter TST, lower sleep efficiency, longer sleep latency, and shorter N3. Contrarily, the negative effect of backward rotation was not greater than that of the LL shift combination. Interestingly, when the LL shift combination became longer, the N3 sleep length decreased by 5 min. Probably, the employees, who worked in the LL shift combination, could not have a sufficiently long off‐duty period between the ‘time to go home’ and ‘bedtime’. Furthermore, a higher level of apprehension for the next working day is associated with a lower amount of PSG‐measured slow wave sleep (Kecklund and Akerstedt 2004). Besides, it is difficult to mentally detach from work within the off‐duty period of a LL shift combination. Given that psychological detachment plays an essential role in the recovery from work (Sonnentag et al. 2010), it is logical that LL shift combination decreases N3 sleep. However, in a previous study, evening (e.g., 8:00 PM–12:00 AM)‐to‐evening shift combination (which is like LL shift in this study) increased the sleep duration (measured by sleep diary logging, which is subjective) in nurses (Vedaa et al. 2017). Despite the differences in the study target populations, this discrepancy in results can be attributed to the differences in the methods employed to measure sleep quality (objective ring‐type sleep tracking vs. subjective diary logging). Specifically, our finding gives deeper insights into shift combination‐induced changes in sleep quality. To the best of our knowledge, the results presented here represent the first objectively measured data on shift combination‐induced changes in sleep quality.

This study has several strengths. First, we used objective measurements of sleep outcomes. Therefore, our data are informative enough to evaluate the effectiveness of the AI‐driven shift‐scheduling app in improving sleep outcomes. Second, the results of our study are more reliable than those obtained using cross‐sectional or observational studies because we used an interventional cross‐over design. Third, the target study population was composed of shift‐working geriatric caregivers. Given that research on shift‐working geriatric caregivers is very limited, the findings of our study will be helpful in improving their health and safety. Third, the collaborating company reported that the app could save 1/12 of working time compared to the manual method of making a shift schedule. The data was not based on this study, but it suggested the possible benefits of this app. Also, since a single user requires approximately three euros (approximately ¥500) monthly, introducing a workplace would not be challenging. However, our study has some limitations. First, the effectiveness of using the AI‐driven shift‐scheduling app is dependent on working characteristics. Specifically, to elaborate tailormade strategies fitting the targeted workplace, we conducted occupational‐fatigue counselling as a participatory ergonomic approach (Kogi 1995; Tsutsumi et al. 2009), since the workplace strategy for reducing fatigue and sleepiness could not be effective without considering workplace characteristics. This approach has positive and negative aspects in terms of theory and practice. Positively, designing interventions using the participatory approach could be more effective in improving the conditions of a targeted workplace. Negatively, the generalisability of the interventional effectiveness obtained here is limited. However, our main finding that reduction of backward shift rotation improves sleep quality, which is in agreement with earlier reports (Sallinen and Kecklund 2010; Garde et al. 2020; Viitasalo et al. 2008), can be adapted to other workplaces. Second, since the average age of participants was 40 years, the quality of sleep measured here cannot be extrapolated to studies with younger populations. Third, this study did not evaluate some factors (e.g., work status, responsibilities outside of the workplace and home) that potentially affect the outcomes. Besides, a validated inventory regarding sleep problems was not used. However, based on the objectively measured sleep data, no participants slept throughout this study period. Fourth, in terms of methodology, we could not control the number of night shifts to put on an equal night shifts condition. Namely, steps should be taken to ensure the homogeneity of participants with respect to the exposure to working night shifts. Besides, we should assign the following three conditions to test the effectiveness of the shift‐scheduler: (1) manual shift scheduling of each workplace, (2) manual shift scheduling with conscious attempts regarding four shift scheduling improvement needs (avoiding backward rotating shift, reducing consecutive shifts, extending shift intervals, and ensuring days‐off after a night shift), (3) AI‐driven shift scheduling with the attempts regarding the four improvement needs. Fifth, we did not conduct a power analysis to determine the sample size because we could not find a similar study using the ring‐type sleep tracker device.

## 5 Conclusion

AI‐driven shift‐scheduling app‐created ‘intervention’ shift schedules had a lower number of backward rotating shifts compared with the manually created shift schedules. Consequently, this study suggests that the intervention condition had longer N3 and REM sleep than the control. However, the generalisability of our findings must be carefully interpreted, since the observed effectiveness of the intervention could be attributable to the participatory ergonomic approach. Therefore, to be effective, the shift interventions suggested here must be refined using occupational‐fatigue counselling, which reflects the localised real‐world improvement needs of employees in other workplaces.

## Author Contributions

**Tomohide Kubo:** conceptualization, data curation, investigation, writing – original draft. **Shun Matsumoto:** conceptualization, data curation, investigation, methodology, writing – review and editing. **Yuki Nishimura:** conceptualization, data curation, methodology, investigation, writing – review and editing. **Hiroki Ikeda:** investigation, methodology, writing – review and editing. **Shuhei Izawa:** investigation, writing – review and editing. **Fumihiko Sato:** conceptualization, data curation, methodology, writing – review and editing.

## Ethics Statement

The Institutional Review Board of the National Institute of Occupational Safety and Health, Japan, reviewed and approved the study protocol (2022N‐1‐6). All participants gave written informed consent.

## Consent

The authors have nothing to report.

## Conflicts of Interest

This study was conducted as collaborative research between National Institute of Occupational Safety and Health, Japan (JNIOSH) and SynCube. However, there was no financial support from SynCube to JNIOSH provided.

## Supporting information


**Figure S1.** Results of the post hoc analysis concerning sleep outcomes with significant main effect [* stands for *p* < 0.05].

**Figure S2.** Results of the post hoc analysis considering psychological outcomes with significant main effect [* stands for *p* < 0.05].

## Data Availability

Data are available upon reasonable request.
