# Supplementary figures and images for: A Participatory Artificial Intelligence Driven Shift‐Scheduling Application for Improving Sleep Among Shift‐Working Caregivers: A 4‐Month Non‐Randomised Controlled Study With Cross‐Over Design
Source: J Sleep Res. 2025 Jul 17;35(1):e70144. doi: 10.1111/jsr.70144 (PMC12856135; doi:10.1111/jsr.70144)

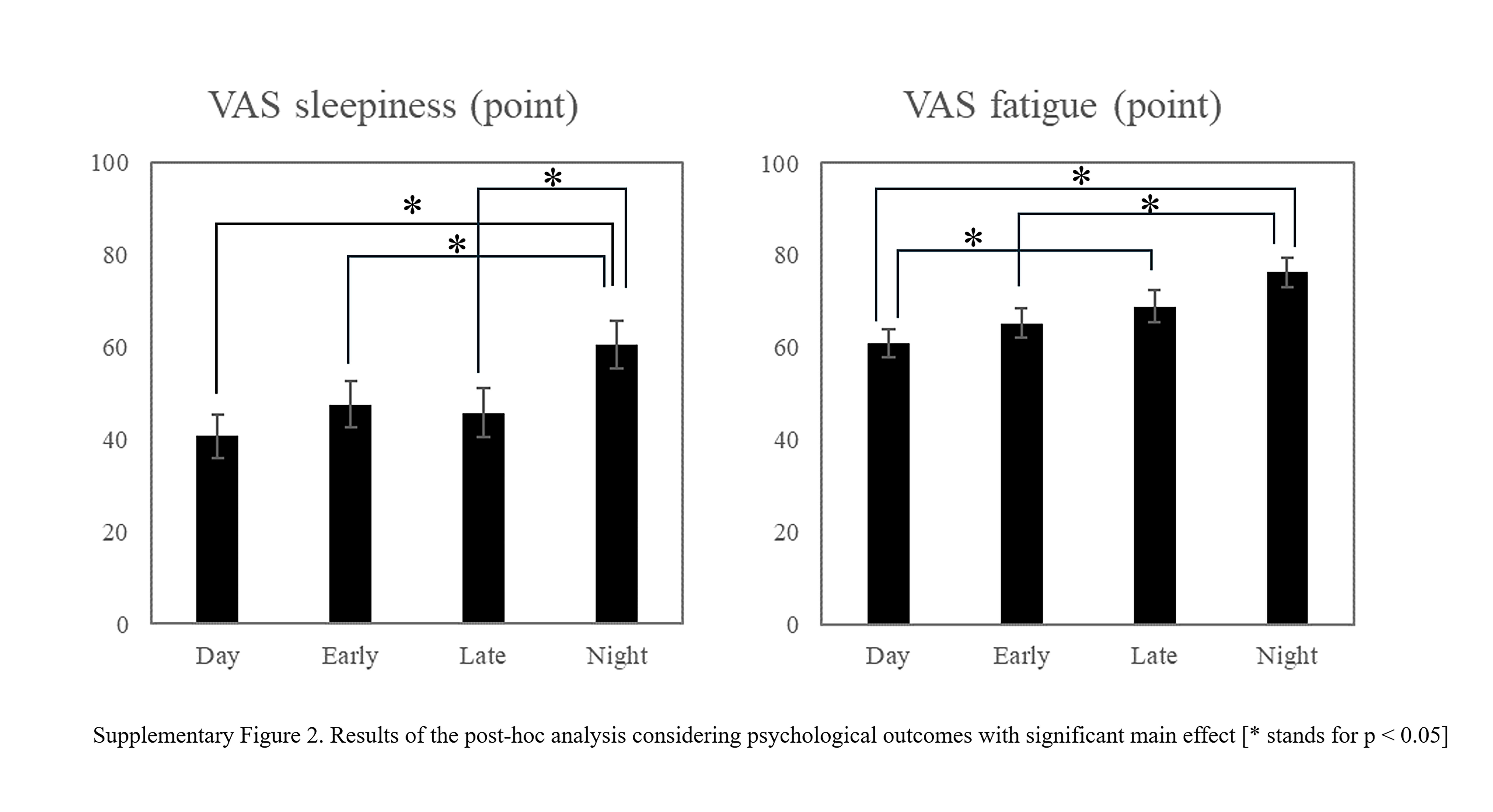

Supplement: Supplementary file 2 — Figure S2. Results of the post hoc analysis considering psychological outcomes with significant main effect [* stands for p < 0.05]. [file JSR-35-e70144-s002.tif]
